# Vitamin B12 in Obese Adolescents with Clinical Features of Insulin Resistance

**DOI:** 10.3390/nu6125611

**Published:** 2014-12-04

**Authors:** Mandy Ho, Jocelyn H. Halim, Megan L. Gow, Nouhad El-Haddad, Teresa Marzulli, Louise A. Baur, Chris T. Cowell, Sarah P. Garnett

**Affiliations:** 1 Institute of Endocrinology and Diabetes, the Children’s Hospital at Westmead, Locked Bag 4001, Westmead, NSW 2145, Australia; E-Mails: mandy.ho@sydney.edu.au (M.H.); jocelyn.halim@gmail.com (J.H.H.); megan.gow@health.nsw.gov.au (M.L.G.); chris.cowell@health.nsw.gov.au (C.T.C.); 2 The Children’s Hospital at Westmead Clinical School, University of Sydney, Locked Bag 4001, Westmead, NSW 2145, Australia; E-Mail: Louise.Baur@health.nsw.gov.au; 3 Centre for Primary Health Care and Equity, University of New South Wales, Sydney, NSW 2052, Australia; E-Mail: nouhadelhaddad@gmail.com; 4 Department of Haematology, The Children’s Hospital at Westmead, Locked Bag 4001, Westmead, NSW 2145, Australia; E-Mail: teresa.marzulli@health.nsw.gov.au; 5 Kids Research Institute, the Children’s Hospital at Westmead, Locked Bag 4001, Westmead, NSW 2145, Australia

**Keywords:** vitamin B12 deficiency, cobalamin, obesity, adolescents, insulin resistance

## Abstract

Emerging evidence indicates an association between obesity, metformin use and reduced vitamin B12 status, which can have serious hematologic, neurologic and psychiatric consequences. This study aimed to examine B12 status in obese adolescents with pre-diabetes and/or clinical features of insulin resistance. Serum B12 was measured using chemiluminescence immunoassay in 103 (43 male, 60 female) obese (mean body mass index (BMI) *z*-score ± SD (2.36 ± 0.29)), adolescents aged 10 to 17 years, median (range) insulin sensitivity index of 1.27 (0.27 to 3.38) and 13.6% had pre-diabetes. Low B12 (<148 pmol/L) was identified in eight (7.8%) and borderline status (148 to 221 pmol/L) in an additional 25 (24.3%) adolescents. Adolescents with borderline B12 concentrations had higher BMI *z*-scores compared to those with normal concentrations (2.50 ± 0.22 *vs.* 2.32 ± 0.30, *p* = 0.008) or those with low B12 concentration (2.50 ± 0.22 *vs*. 2.27 ± 0.226, *p* = 0.041). In conclusion, nearly a third of obese adolescents with clinical insulin resistance had a low or borderline serum B12 status. Therefore, further investigations are warranted to explore the cause and the impact of low B12 status in obese pediatric populations.

## 1. Introduction

Vitamin B12, an essential vitamin found in animal and fortified food products, has a fundamental role in DNA synthesis, optimal haemopoiesis and neurological function. B12 deficiency is associated with a spectrum of disease from asymptomatic to serious haematological, neurologic and psychiatric manifestations and the possible risk of irreversible neurological damage despite treatment [1]. B12 deficiency is well documented in adults with inadequate intake, gut malabsorption or pernicious anaemia. Malabsorption of B12 is also associated with metformin therapy, an insulin sensitizer used for the treatment of type 2 diabetes [2] and increasingly in obese, insulin resistant, adolescents [3].

Results from two recent studies also indicate that low B12 concentrations may be associated with obesity during childhood [4,5]. Pinhas-Hamiel *et al.* [5] reported a greater than 4-fold increased risk of low vitamin B12 status in obese compared to normal weight Israeli children and adolescents. The report from the population based Canadian Health Measurement Survey indicated that obese 6 to 19 year olds were more likely to have inadequate vitamin B12 status compared to individuals with a normal weight [4]. However, significant associations are not consistently reported [6]. To our knowledge, B12 status in adolescents with pre-diabetes and/or clinical feature of insulin resistance, a population at increased risk of type 2 diabetes, has not been examined. Nevertheless the high prevalence of childhood obesity, long-term metformin therapy for those with or at risk of type 2 diabetes, and the potential severity vitamin B12 deficiency has on health, provides merit for further research. Hence, this study aimed to examine B12 status in adolescents with pre-diabetes and/or clinical features of insulin resistance.

## 2. Experimental Section

### 2.1. Subjects

Participants were 10 to 17 year old adolescents (43 male, 60 female) with pre-diabetes and/or clinical features of insulin resistance enrolled in a 12-month randomised controlled trial called RESIST (ACTRN12608000416392). The RESIST protocol and inclusion/exclusion criteria have been previously published [7,8]. In brief, clinical features of insulin resistance were defined as fasting insulin (U/L)/glucose (mmol/L) ratio >20 with one or more of the following: acanthosis nigricans, polycystic ovarian syndrome, hypertension, or dyslipidaemia. The study was approved by The Children’s Hospital at Westmead Human Research Ethics Committee (07/CHW/12). Written informed consent from parents and assent from the participants was given prior to enrolment. Baseline measurements were used for this analysis, none of the participants was taking metformin.

### 2.2. Assessment

Weight and height were measured, body mass index (BMI) calculated and z-scores and weight status (using the International Obesity Task Force criteria) determined. Body composition was measured by dual-energy X-ray absorptiometry (Prodigy, Lunar-GE, Madison, WI, USA) equipped with proprietary software version 13.6. Insulin sensitivity index (ISI) was derived from an oral glucose tolerance test using the Matsuda formula [9]. Serum B12 concentrations were measured using a chemiluminescence immunoassay (Access Immunoassay, Beckman Coulter, Fullerton, CA, USA) and categorised as low (<148 pmol/L), borderline (148–221 pmol/L), and normal (>221 pmol/L) [4]. Serum folate and red blood cell (RBC) folate were analyzed by using an immunoassay analyser (Elecsys Folate Assay, Roche Diagnostics, Mannheim, Germany) and categorised as low (<320 nmol/L), adequate (320–1090 nmol/L), and high RBC folate (>1090 nmol/L) [4]. Blood parameters were determined using the automated full blood count analyzer (DxH800, Beckman Coulter, Fullerton, CA, USA). Study physicians assessed presence of acanthosis nigricans. Blood pressure was measured using an automated blood pressure (BP) monitor (Dinamap 1846 SX, GE Healthcare, Indianapolis, Indiana) according to standard procedures. The mean of three replicates were used for analysis. Elevated BP was defined as the 90th percentile or greater, using age-, height-, and sex-specific reference values [10]. Lipid profile was determined using blood samples collected in the morning after an overnight fast. Adolescents with elevated levels of triglycerides ≥1.7 mmol/L and/or high-density lipoprotein cholesterol <1.03 mmol/L were identified as having dyslipidaemia.

### 2.3. Statistics

Data were assessed for normality and analysed using the SPSS Statistical Package, version 19.0 (SPSS Inc., Chicago, IL, USA). Consistent with data distribution, differences between B12 status groups were assessed by analysis of variance (ANOVA) or Kruskal-Wallis. χ^2^ tests were used as a measure of association between categorical variables.

## 3. Results

**Figure 1:**
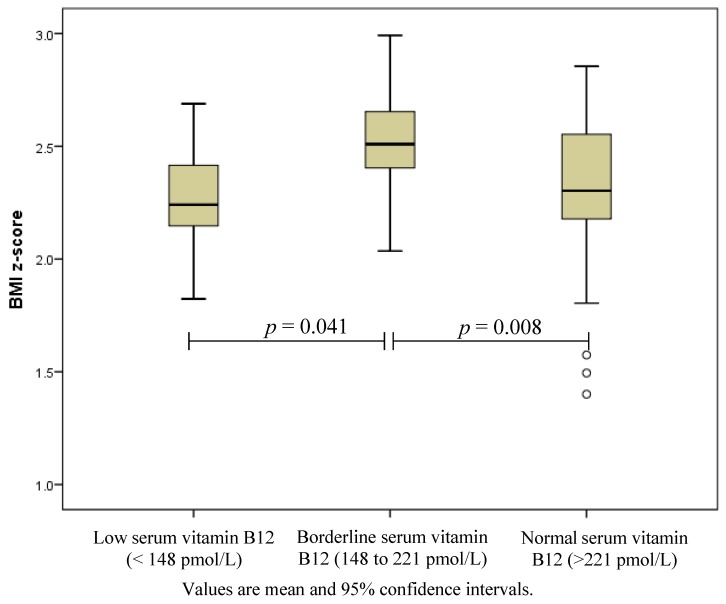
Body mass index *z*-scores of adolescents with low (*n* = 8), borderline (*n* = 25) and normal (*n* = 70) serum B12 concentrations.

The median (range) age of participants was 13.4 (10.1 to 17.4) years. Most were obese (96.1%) and presented with acanthosis nigricans (84.5%), elevated BP (45.6%) and/or dyslipidaemia (54.4%). Median insulin sensitivity index (derived from an oral glucose tolerance test using the Matsuda formula) was 1.27 (0.27 to 3.38), and 13.6% participants were pre-diabetic. Serum B12 were median (range) 255 (102 to 606) pmol/L. Eight (7.8%) adolescents had low and an additional 25 (24.3%) had borderline B12 status. There was no statistical difference in age, sex, total body fat%, glycaemic status, BP status, serum folate, haemoglobin concentration or mean corpuscular volume between adolescents in the different B12 status groups, Table 1. However, adolescents with serum vitamin B12 concentrations ≤221 pmol/L had a higher BMI *z*-score than those with normal concentrations, *p* = 0.047 (Figure 1). There was a greater, but non-significant, proportion who had low/borderline B12 concentrations with macrocytosis (mean corpuscular volume > 85 fl/L), 21% *vs.* 11%, *p* = 0.189.

Serum folate and RBC folate concentrations were median (range) 29.5 (4.6 to 45.3) nmol/L and 840 (429 to 2000) nmol/L, respectively. None of the adolescents had low RBC folate (<320 nmol/L), though 18 (17%) adolescents had high RBC folate status, median 1238, (1091 to 2000) nmol/L. The median RBC folate concentrations were significantly lower in adolescents with low B12 concentrations than adolescents with normal B12 concentrations (*p* = 0.012). There was no statistical difference in sex (*p* = 0.185), glycaemic status (*p* = 0.915), BP status (*p* = 0.532), blood lipids status (*p* = 0.397) and BMI z-scores (*p* = 0.421) between adolescents with normal and adolescents with high RBC folate concentration. However, adolescents who had high RBC folate concentration had higher total body fat% (49.3 ± 5.65 *vs.* 46.4 ± 4.59, *p* = 0.004) than those had a normal RBC folate concentration. Furthermore, there were a greater proportion of adolescents who had high RBC folate concentration were pre-pubertal (36% *vs.* 11%, *p* = 0.004). All adolescents with macrocytosis had a normal RBC folate concentration.

**Table 1:** Body composition, insulin sensitivity and blood profile of participants (*n* = 103) at baseline. Values are mean ± SD unless otherwise indicated. BMI, body mass index; RBC, red blood cell.

	Low B12 < 148 pmol/L *n* = 8	Borderline B12 148 to 221 pmol/L *n* = 25	Normal B12 > 221 pmol/L *n* = 70	*P*-Value *
Age (years)	12.7 ± 1.6	13.2 ± 1.8	13.4 ± 1.9	0.610
Overweight/obese ^†^	0/8	0/25	4/66	0.198
Male *n* (%)	2 (25)	9 (36)	32 (46)	0.260
Weight (kg)	84.2 ± 18.0	99.3 ± 18.3	90.7 ± 20.0	0.083
BMI	31.8 ± 5.8	37.1 ± 5.	33.7 ± 5.0	0.008
BMI z-score	2.27 ± 0.26	2.50 ± 0.22	2.32 ± 0.30	0.017
Fat mass % ^‡^	50.4 ± 5.3	50.1 ± 4.8	48.2 ± 5.9	0.296
Insulin sensitivity index ^§^	1.18 (0.53 to 3.38)	1.37 (0.27 to 3.03)	1.34 (0.30 to 3.34)	0.853
Pre-diabetes ^¶^ *n* (%)	2 (25)	4 (16)	8 (11)	0.563
B12 pmol/L median (range)	120 (102 to 140)	196 (151 to 218)	296 (222 to 606)	<0.001
Serum folate nmol/L median (range)	23.4 (15.9 to 45.3)	28.9 (4.6 to 45.3)	29.9 (6.3 to 45.3)	0.583
RBC folate nmol/L median (range) ^††^	596 (465 to 1150)	811 (429 to 1240)	860 (466 to 2000)	0.036
Haemoglobin g/L ^#^	130 ± 13	136 ± 10	135 ± 10	0.352
Low haemoglobin <95 g/L *n*	0	0	0	
Hematocrit % ^#^	38 ± 3	40 ± 2	40 ± 3	0.238
<28% *n*	0	0	0	
Mean corpuscular volume fL ^#^ >85 fL *n* (%)	82 ± 4 1 (13)	82 ± 4 6 (24)	81 ± 4 8 (11)	0.658 0.306

^†^ Obesity defined by International Obesity Taskforce [11]; ^‡^ one adolescent did not have fat mass measured; ^§^ derived from Matsuda and DeFronzo [9]; ^¶^ pre-diabetes defined by American Diabetes Association [12]; ^††^ two adolescents did not have RBC folate measured; ^#^ normal ranges at The Children’s Hospital at Westmead for haemoglobin = 95 to 140 g/L, hematocrit = 28%–45%, and mean corpuscular volume = 70 to 85 fL; * *p*-values for on-way ANOVA test (normally distributed data), Kruskal-Wallis (non-parametric data) and χ^2^ tests (categorical data).

Values are mean and 95% confidence intervals.

## 4. Discussion

To our knowledge this is the first study to examine vitamin B12 status in adolescents with pre-diabetes and/or clinical features of insulin resistance. The results indicate that almost one-third of the obese adolescents were classified with low or borderline vitamin B12 status. The percentage of adolescents identified with low or borderline B12 status in our study (32.1%) is greater than the 13.7% (all children and adolescents) and 20.4% (obese children and adolescents) reported in a Canadian population-based survey using the same cut-points [4]. We speculate that obese adolescents at risk of developing type 2 diabetes may have a greater risk of low B12 status compared to the general paediatric obese population. This is of particular concern for these adolescents because they are susceptible to a further decrease in vitamin B12 concentrations if metformin therapy is commenced [2]. In adults, vitamin B12 malabsorption occurs in approximately 10%–30% of patients using metformin and is associated with a 4%–24% decrease of vitamin B12 [13].

Consistent with previous studies [4,5], adolescents with serum vitamin B12 concentrations ≤221 pmol/L had higher BMI *z*-scores with no significant sex difference. Reduced vitamin B12 concentrations in the paediatric obese population is thought to result from insufficient intake from a nutrient poor diet, repeated short-term restrictive diets and/or increased nutrient requirements secondary to increased growth and body size [5]. Vitamin B12 plays an important role in the DNA methylation. A recent genome-wide analysis suggested that increased DNA methylation is associated with increased BMI in adults [14]. The lack of association of serum vitamin B12 with total body fat % or ISI in this study may be attributed to the high body fat proportion of all the adolescents in our cohort.

There is no consensus on diagnostic guidelines to determine B12 deficiency. We measured serum B12, which is the most accessible and cost effective diagnostic test, but lacks universally accepted cut-points [15]. The cut-points of 148 pmol/L and 221 pmol/L used in this study are reported to have a sensitivity of 95%–97% and 99%, respectively [15,16]. Using these criteria it is unlikely that we have overestimated the prevalence of low/borderline B12 concentrations on B12 status. However, we did not measure holotranscobalamin (active B12) and metabolic markers (methylmalonic acid and homocysteine) which may be a more sensitive indicator of B12 status. Another limitation of current study is that we did not have a control group of normal weight adolescents. Furthermore, the cross-sectional design of the study represents a limitation of causality cannot be established. The clinical interpretation of our findings is unclear and impact of observed low/borderline B12 is unknown. Macrocytosis was observed in 15% of adolescents with low/borderline B12; this may be a normal variation or an early marker of future anaemia. The strength of current study is that we included the analysis of RBC folate status. The metabolism of B12 and folate is interdependent. A high folate status can mask the haematological symptoms of B12 deficiency and exacerbate the clinical symptoms of B12 deficiency, including elevated plasma homocysteine, a biomarker of many chronic diseases. We did not find an association between high RBC folate status and low serum B12 status. In contrast, obese adolescents with low serum B12 status showed a lower RBC folate concentration than adolescents with normal B12 concentration. Replication of these findings in a larger sample with the inclusion of dietary intake data is warranted to explore the cause and the impact of low B12 status in the obese pediatric population.

## 5. Conclusions

In conclusion, the substantial number of obese adolescents at risk of type 2 diabetes identified with a low or borderline B12 status, and the potential severity of B12 deficiency indicates that the association between obesity, insulin resistance, metformin use and low B12 status warrants further research in this population.
